# Fluoxetine Is Associated with Reduced Cell Viability and Decreased pERK1/2 Immunoreactivity in Ishikawa Endometrial Cancer Cells In Vitro: An Exploratory Study

**DOI:** 10.3390/life16101629

**Published:** 2026-09-29

**Authors:** Neziha Senem Arı, Ayşe Çakır Gündoğdu, Neslihan Meriç, Orhan Özatik

**Affiliations:** 1 Department of Histology and Embryology, Faculty of Medicine, Kütahya Health Sciences University, 43100 Kutahya, Türkiye; 2 Department of Molecular Biology and Genetics, Faculty of Engineering and Life Sciences, Kütahya Health Sciences University, 43100 Kutahya, Türkiye

**Keywords:** fluoxetine, drug repurposing, selective serotonin reuptake inhibitor, Ishikawa cell line, endometrial carcinoma, MAPK/ERK pathway, ERK1/2 phosphorylation, in vitro cytotoxicity, TUNEL, annexin V/propidium iodide, exploratory study

## Abstract

Selective serotonin reuptake inhibitors are widely prescribed in oncology, yet their direct effects on endometrial cancer cell fate remain unclear. This study examined whether fluoxetine, at concentrations referenced to reported plasma exposure ranges, alters viability, cell death phenotype, DNA integrity and extracellular signal-regulated kinase 1/2 (ERK1/2) signalling. Ishikawa endometrial cancer cells and normal human dermal fibroblasts (HDFs) were exposed to fluoxetine (100–300 ng/mL) for a single exposure period of 48 h. Metabolic viability was assessed by MTT assay, cell death phenotypes by annexin V/propidium iodide (PI) flow cytometry, DNA fragmentation by TUNEL, and total and phosphorylated ERK1/2 (pERK1/2) by immunofluorescence; all experiments were performed in three independent experiments (*n* = 3), and per-experiment means were used as the unit of analysis. Fluoxetine reduced metabolic viability in Ishikawa cells, with significant reductions detected at 100 and 300 ng/mL, whereas HDF viability remained largely preserved. Flow cytometry showed predominantly viable populations in both cell types, with low early apoptotic, late apoptotic and annexin V^−^/PI^+^ fractions; no statistically significant treatment-related differences were detected in these cell death-associated populations, although analysis at a single 48 h endpoint cannot exclude earlier transient changes. TUNEL positivity showed a concentration-related numerical increase in Ishikawa cells, reaching statistical significance at 300 ng/mL, while remaining low in HDF cells. pERK1/2 immunoreactivity was significantly decreased at 300 ng/mL in both Ishikawa and HDF cells without a comparable change in total ERK1/2 immunoreactivity; because this readout was obtained by immunofluorescence only and was not validated by Western blotting, it is reported as a change in immunoreactivity rather than as confirmed pathway inhibition. These exploratory in vitro findings indicate that fluoxetine exposure is associated with reduced viability, DNA fragmentation and decreased pERK1/2 immunoreactivity in Ishikawa cells; the limited injury in HDF cells reflects a general non-malignant comparator response rather than cancer-specific selectivity. The findings are hypothesis-generating and require confirmation by protein- and transcript-level validation, pathway modulation experiments and additional malignant and non-malignant cell models.

## 1. Introduction

Endometrial cancer is among the most frequently diagnosed gynaecological malignancies and remains a significant cause of cancer-related morbidity and mortality worldwide. Although early-stage disease is often associated with favourable clinical outcomes, advanced and recurrent forms continue to pose substantial therapeutic challenges [1]. The biological behaviour of endometrial tumours is governed by a complex interplay of hormonal regulation, growth factor signalling and intracellular stress-response pathways, all of which collectively shape cellular decisions related to proliferation, survival and death. These signalling networks do not operate in isolation; rather, they dynamically interact to determine whether a cell adapts to stress, enters a quiescent state, or undergoes irreversible death. Understanding how pharmacological agents perturb these networks is therefore critical for identifying novel vulnerabilities in cancer cells and for improving therapeutic strategies [2].

In parallel with the oncological burden of the disease, psychological distress and depressive symptoms are highly prevalent among patients with cancer. Depression not only compromises quality of life but also negatively influences treatment adherence, patient engagement and overall clinical outcomes. As a result, antidepressant therapy is frequently prescribed to individuals undergoing cancer treatment [3]. Among available antidepressants, selective serotonin reuptake inhibitors (SSRIs) are widely used due to their favourable safety profiles and broad clinical efficacy. Fluoxetine, one of the earliest and most extensively prescribed members of this class, has been used for decades in the management of mood disorders. While its primary mechanism of action involves modulation of serotonergic neurotransmission, it is increasingly recognised that fluoxetine also exerts direct biological effects on non-neuronal cells, including immune cells, endothelial cells and a variety of tumour cell types [4,5,6].

This growing recognition has prompted a shift in perspective, whereby antidepressants are no longer viewed solely as neuropsychiatric agents but also as potential modulators of tumour cell biology. Preclinical evidence, including in vitro studies across multiple cancer models (e.g., non-small cell lung cancer, triple-negative breast cancer, bladder cancer and osteosarcoma) and in vivo validation in some settings, suggests that fluoxetine can influence cell proliferation, oxidative balance, mitochondrial function and regulated cell death programmes [4,7,8,9,10]. However, the reported effects are not uniform; rather, they appear to be highly context-dependent, varying with cell type, concentration and duration of exposure. In some systems, fluoxetine has been reported to suppress proliferation and induce apoptosis, whereas in others it appears to promote cellular stress responses that may or may not culminate in cell death. This heterogeneity underscores the necessity of tumour-type-specific investigations and highlights the importance of pathway-based studies that move beyond descriptive cytotoxicity assays [5].

A central signalling axis that warrants particular attention in this context is the mitogen-activated protein kinase/extracellular signal-regulated kinase (MAPK/ERK) pathway. ERK1/2 integrates signals from a broad range of extracellular stimuli, including growth factors, hormones and stress cues, and translates them into transcriptional programmes that regulate proliferation, differentiation, survival and resistance to therapy [11]. ERK1/2 is activated through dual phosphorylation of the threonine and tyrosine residues within the conserved Thr-Glu-Tyr (TEY) motif in its activation loop; therefore, pERK1/2 levels are widely used as a practical readout of ERK pathway activation. Activated ERK1/2 can phosphorylate cytosolic substrates and translocate to the nucleus, where it influences transcription factor activity and reshapes gene expression programmes linked to proliferation, survival and stress responses [12]. Aberrant ERK signalling is a hallmark of many malignancies and has been implicated in tumour progression, metastatic potential and treatment resistance [13]. In endometrial cancer cell models, AF-6/afadin knockdown significantly increased Matrigel invasion in Ishikawa and HEC1A cells; in Ishikawa cells, this enhanced invasiveness was accompanied by increased phosphorylation of ERK1/2 and Src [14].

In endometrial cancer, ERK signalling occupies a particularly prominent role. Ishikawa cells, a well-established in vitro model of endometrioid endometrial carcinoma, exhibit strong hormonal responsiveness and active cross-talk between oestrogen receptor signalling, phosphoinositide 3-kinase/AKT pathways and MAPK/ERK cascades. Within this network, ERK1/2 functions not merely as a downstream effector of mitogenic signalling but also as a regulator of cellular survival and apoptotic signalling. Perturbations in ERK phosphorylation can therefore shift the balance between adaptive responses and cell death [15]. Three considerations motivated the specific focus on ERK1/2 in the present study. First, in other tumour models the anti-proliferative effects of fluoxetine have repeatedly been linked to modulation of ERK- and STAT3/NF-κB-associated survival signalling, which makes ERK1/2 the candidate node most consistently implicated for this particular drug [8,9,10,16]. Second, in Ishikawa cells ERK1/2 lies at the convergence point of oestrogen receptor, PI3K/AKT and growth factor inputs, so a change in its activation state can be read as an integrated indicator of survival signalling rather than as an isolated event [14,15]. Third, ERK1/2 activation is defined by a discrete, phosphorylation-dependent readout that can be quantified in situ at the level of individual cells, which is compatible with the sub-cytotoxic exposure window used here. ERK1/2 was therefore selected a priori as the primary signalling readout of this study rather than identified post hoc among several screened pathways. Evaluating how fluoxetine modulates ERK1/2 activation in Ishikawa cells provides a pathway-level entry point into understanding whether this antidepressant reshapes survival signalling in endometrial cancer.

A further methodological consideration concerns dose selection. Many in vitro studies of repurposed drugs employ concentrations that greatly exceed clinically achievable levels, which can obscure biologically relevant mechanisms and favour non-specific cytotoxicity [17]. In contrast, selecting experimental concentrations with reference to reported plasma exposure ranges increases the likelihood that observed effects reflect plausible biological interactions rather than generalised toxicity. In addition, the inclusion of a non-malignant comparator cell type provides an initial comparison of responses in malignant and general non-malignant cells and provides insight into whether drug effects preferentially target cancer cells or extend indiscriminately to normal cells.

Accordingly, this study was designed to systematically evaluate the in vitro effects of fluoxetine on Ishikawa endometrial cancer cells, focusing on cell viability, cell death phenotype, DNA fragmentation and ERK1/2 signalling activity within a sub-cytotoxic concentration range. Despite a growing body of literature suggesting an antitumour potential of antidepressants across different cancer models, data addressing the effects of fluoxetine in endometrial cancer cells—particularly in relation to ERK1/2 phosphorylation—remain limited; therefore, the present study aims to fill this knowledge gap [6]. By including normal human dermal fibroblasts as a general non-malignant reference, we sought to obtain an initial indication of non-malignant cell tolerance rather than to establish endometrial cancer-specific selectivity. The integrated assessment of metabolic viability, flow cytometry-based cell death phenotyping, TUNEL and ERK1/2 phosphorylation/total protein levels is expected to provide a more comprehensive view of how fluoxetine exposure is associated with survival- and stress-response-related signalling changes in endometrial cancer cells. Because the design employs a single endometrial cancer cell line, a single non-malignant comparator and a single exposure time point, and because the signalling readout is based on immunofluorescence without immunoblot confirmation, the study is explicitly framed as an exploratory, hypothesis-generating investigation rather than as a mechanistic or confirmatory one.

## 2. Materials and Methods

### 2.1. Cell Culture and Maintenance

Human endometrial adenocarcinoma Ishikawa cells and normal adult human dermal fibroblasts (HDFs) were used as malignant and non-malignant cellular models, respectively. The Ishikawa cell line originated from the European Collection of Authenticated Cell Cultures (ECACC, Salisbury, UK; catalogue number 99040201; RRID:CVCL_2529). HDF cells are commercially available primary adult human dermal fibroblasts obtained from the American Type Culture Collection (ATCC, Manassas, VA, USA; catalogue number PCS-201-012); because they are primary cells rather than an established immortalised line, no cell line database accession number is applicable. The cell stocks used in the present study originated from the commercial collections specified above.

Ishikawa cells were cultured in RPMI-1640 medium (Gibco, Thermo Fisher Scientific, Waltham, MA, USA), while HDF cells were maintained in Dulbecco’s Modified Eagle Medium (DMEM; Gibco). All media were supplemented with 10% foetal bovine serum (FBS), 1% L-glutamine and penicillin (100 U/mL)–streptomycin (100 µg/mL) to provide optimal growth conditions and prevent microbial contamination.

Cells were incubated at 37 °C in a humidified atmosphere containing 5% CO_2_. Routine subculturing was performed using trypsin–EDTA solution once the cells reached approximately 70–80% confluence. For experimental procedures, cells were seeded into 96-well, 24-well or 6-well culture plates depending on the specific assay requirements. All experiments were conducted using cells within a limited passage range (Ishikawa, passages 5; HDF, passages 4) to minimise phenotypic drift and, in the case of the primary HDF cultures, replicative senescence.

### 2.2. Drug Preparation and Dose Selection

Fluoxetine hydrochloride with ≥95% purity was obtained from abcr GmbH (Karlsruhe, Germany; product code AB 401002). Fresh 1 mM stock solutions were prepared immediately prior to each independent experiment by dissolving fluoxetine hydrochloride directly in the appropriate culture medium for each cell line (RPMI-1640 for Ishikawa cells and DMEM for HDF cells) and were sterilised by filtration through a 0.22 µm membrane filter. No organic co-solvent was used during stock preparation. The stock solutions were diluted in complete culture medium immediately before addition to the cells to obtain final fluoxetine concentrations of 100, 200 and 300 ng/mL. The fluoxetine-containing medium was applied once and was not renewed during the 48 h exposure period; therefore, the reported concentrations represent the nominal concentrations at the beginning of treatment. The chemical stability of fluoxetine and its residual concentration in the culture medium over the 48 h exposure period were not verified analytically in the present study; this is acknowledged as a limitation in Section 4.

Dose selection was performed using a stepwise, data-driven approach. In the initial phase, a broad concentration range was determined based on previously published in vitro studies evaluating the biological and anti-proliferative effects of fluoxetine in different cancer cell lines; these studies reported that fluoxetine was generally applied at micromolar levels (approximately 5–40 µM) [6,16]. Accordingly, cells were exposed to 5, 10, 15, 20, 25, 30, 35, 40, 45 and 50 µM fluoxetine hydrochloride for 72 h, and the dose–response profile was evaluated for preliminary screening. In these preliminary screening experiments, rapid loss of membrane integrity developed in cells, particularly at high micromolar concentrations, and a marked increase in the annexin V^−^/propidium iodide (PI)^+^ cell population was observed in flow cytometry analyses. These findings indicated that cell death at these concentrations predominantly exhibited necrotic characteristics and that early apoptotic responses were limited. Considering that excessive necrotic cell death could mask the evaluation of specific cellular signalling pathways and regulatory biological responses, the experimental approach was reconfigured in the second phase. In this phase, dose selection was based on the steady-state (C_ss_) plasma concentrations reported for chronic fluoxetine use, and experimental concentrations were created using a 0.1×, 1× and 10× multiplier approach. The steady-state/plateau concentrations of fluoxetine reported in human plasma after chronic use are approximately 91–302 ng/mL [18]. In this context, cells were exposed to fluoxetine hydrochloride concentrations of 25, 50, 100, 150, 200, 250, 300, 600, 900 and 1200 ng/mL for 48 h.

Based on the results obtained, doses of 100, 200 and 300 ng/mL—which fall within the reported steady-state plasma concentration range and allowed the evaluation of cellular responses without causing extensive cytotoxicity—were selected for further experiments. Half-maximal and quartile inhibitory concentrations were estimated in GraphPad Prism v8.4.2 by fitting the normalised MTT dose–response data to a four-parameter log(inhibitor)-versus-response model with variable slope; on this basis, 300 ng/mL corresponded to approximately the IC_25_ level in the present experimental model. This approach allowed the evaluation of sub-cytotoxic biological effects in Ishikawa cells while enabling the preservation of cell viability in non-malignant HDF cells, thereby providing an initial experimental window for comparing responses between Ishikawa and HDF cells.

### 2.3. Cell Viability Assay

The effects of fluoxetine on cell viability were evaluated using the MTT (3-(4,5-dimethylthiazol-2-yl)-2,5-diphenyltetrazolium bromide) assay in Ishikawa endometrial cancer cells and HDF cells. The MTT assay is a widely used colorimetric method for determining cell viability. It is based on the principle that metabolically active cells reduce the yellow MTT to insoluble purple formazan crystals via mitochondrial dehydrogenase enzymes. The amount of formazan formed was used as an indirect indicator of cellular metabolic activity and metabolic viability rather than as a direct measure of cell number.

For MTT analysis, cells were seeded into 96-well plates at a density of 5 × 10^3^ cells per well and allowed to adhere overnight. The MTT assay was performed in three independent experiments conducted on separate days using independently seeded cultures. Within each independent experiment, every experimental condition was assessed in six replicate wells, which were treated as technical replicates. Cells were treated with fluoxetine at concentrations of 100–300 ng/mL. MTT solution (10% *v*/*v*) was added to each well, and cells were incubated at 37 °C for 4 h. After incubation, the solution was carefully removed, and 100 µL of dimethyl sulfoxide (DMSO) was added to each well to dissolve the formazan crystals. The plates were shaken on a heated shaker for 10 min to ensure complete dissolution of the crystals [19].

The absorbance of the resulting formazan dye was measured at 570 nm using a Multiskan™ FC Microplate Photometer (Thermo Fisher Scientific, Waltham, MA, USA). The optical density values obtained from the wells treated with fluoxetine were normalised to the control group and calculated as the percentage of viable cells. The six technical replicate values were averaged within each independent experiment, and the resulting per-experiment means were used as the biological units for statistical analysis (*n* = 3).

### 2.4. Cell Death-Related Pattern Analysis by Flow Cytometry (Annexin V–FITC/PI)

Cell death-related patterns were evaluated by flow cytometry using the annexin V–FITC/PI double staining method. The eBioscience Annexin V-FITC Apoptosis Kit (Thermo Fisher Scientific, Waltham, MA, USA; cat. no. BMS500FI-100) was used for staining according to the manufacturer’s instructions. For analysis, cells were seeded at 5 × 10^5^ cells/well into 6-well plates and allowed to adhere overnight before being treated with fluoxetine at concentrations of 100, 200 and 300 ng/mL and incubated for 48 h. At the end of the treatment, both adherent and suspended cells were collected together, cell pellets were obtained by centrifugation, and the cells were resuspended in 500 µL of 1× binding buffer. For staining, 2 µL annexin V–FITC and 1 µL PI were added to the cell suspensions; samples were incubated at room temperature in the dark for 5 min. Data were acquired using a CytoFLEX flow cytometer (Beckman Coulter Life Sciences, Brea, CA, USA) and analysed using CytExpert software, version 2.5.0.77 (Beckman Coulter Life Sciences), and at least 1 × 10^4^ events were recorded for each sample. Data were evaluated on dot plots, and cell populations were classified as live (annexin V^−^/PI^−^), early apoptotic (annexin V^+^/PI^−^), late apoptotic or secondary membrane-compromised (annexin V^+^/PI^+^), and PI-positive/membrane-compromised (annexin V^−^/PI^+^); results were reported as the percentage distribution of these populations. Quadrant gates were set using unstained, annexin V-only and PI-only single-stain controls. Flow cytometry was performed in three independent experiments conducted on separate days using independently seeded cultures. For each cell population, the values obtained within each independent experiment were summarised, and the resulting per-experiment values were used as the biological units for statistical analysis (*n* = 3).

### 2.5. TUNEL-Based DNA Fragmentation Analysis

DNA fragmentation was evaluated using the terminal deoxynucleotidyl transferase dUTP nick-end labelling (TUNEL) method with the One-step TUNEL In Situ Apoptosis Kit (Red, Elab Fluor 594; Elabscience, Houston, TX, USA; cat. no. E-CK-A322) according to the manufacturer’s instructions. After the relevant procedures, the cells were washed with phosphate-buffered saline (PBS) and fixed with 4% paraformaldehyde at room temperature for 20 min, followed by permeabilisation with 0.1% Triton X-100 for 15 min. Subsequently, the cells were incubated with the kit’s TUNEL reaction mixture in a humid chamber at 37 °C for 30 min. The cells were washed with PBS, and a mounting medium containing DAPI (Sigma-Aldrich, St. Louis, MO, USA; F6057) was used for nuclear counterstaining and sealing the preparations. As a negative control, the TdT enzyme was omitted from the reaction mixture according to the kit protocol, and the procedure was performed under identical conditions. Fluorescent images were acquired at 20× magnification using constant microscope settings and exposure times for all groups.

For quantitative evaluation, five randomly selected fields from each slide were examined under blinded conditions. Cell counts were performed using ImageJ/Fiji v1.52 (National Institutes of Health, Bethesda, MD, USA). TUNEL-positive cells were identified using the same fluorescence thresholding criteria across all groups, and fields with poor focus, excessive cell overlap or edge artefacts were excluded from analysis. Because TUNEL positivity is not specific to apoptosis and may also label necrotic and late-apoptotic nuclei [20,21], the readout is reported here as a TUNEL positivity index (TPI) rather than as an apoptotic index. The TPI was calculated as the percentage of TUNEL-positive cells relative to the total number of DAPI-positive nuclei: TPI (%) = (TUNEL-positive cell count/total DAPI-positive nucleus count) × 100.

### 2.6. ERK1/2 and Phospho-ERK1/2 Immunofluorescence Analysis

Ishikawa and HDF cells were seeded onto 13 mm sterile glass coverslips placed in each well of 24-well culture plates and incubated overnight to ensure adequate cellular adhesion. The next day, the cells were treated at the specified experimental doses for 48 h. After treatment, the cells were washed with PBS (Gibco, 10010-023) and fixed with 4% paraformaldehyde (Sigma-Aldrich, P6148) at room temperature for 20 min. Cell membrane permeabilisation was then achieved by treatment with 0.1% Triton X-100 (Sigma-Aldrich, T8787) for 15 min; blocking was performed with 3% bovine serum albumin (Sigma-Aldrich, A9647) for 30 min to reduce non-specific binding.

Primary antibody incubation was performed overnight at 4 °C using rabbit polyclonal anti-ERK1/2 (Affinity Biosciences, Cincinnati, OH, USA; cat. no. AF0155) and rabbit polyclonal anti-phospho-ERK1/2 (pERK1/2; Thr202/Tyr204; Affinity Biosciences, cat. no. AF1015), both at a 1:200 dilution. Total ERK1/2 and pERK1/2 staining were performed on separate preparations under the same conditions to prevent potential signal overlap. The following day, after sufficient washing with PBS, the cells were incubated at room temperature for 1 h with Alexa Fluor 594-conjugated goat anti-rabbit IgG (Jackson ImmunoResearch, West Grove, PA, USA; 111-585-003) as the secondary antibody. A mounting medium containing DAPI (Sigma-Aldrich, F6057) was used for nuclear counterstaining. For the negative control, the same staining procedure was performed using only the secondary antibody, without applying the primary antibodies. Imaging was performed using a Zeiss Axio Calibri 7 fluorescence microscope (Carl Zeiss, Oberkochen, Germany). Image acquisition was performed under identical exposure, gain, illumination and magnification settings for all groups within each staining set.

Quantitative analysis of fluorescence intensity was performed using ImageJ/Fiji v1.52 by an observer blinded to group allocation, applying identical acquisition and thresholding settings to all groups. Individual cells were manually outlined based on DAPI-positive nuclei and corresponding cellular boundaries, while background fluorescence was measured from cell-free regions of the same image. Corrected total cell fluorescence (CTCF) was calculated as: integrated density − (area of selected cell × mean background fluorescence). At least 50 cells were analysed per group within each independent experiment, and the mean CTCF of each independent experiment was used as the unit of statistical analysis. Fields with overlapping cells, excessive debris or uneven staining were excluded from analysis. It should be noted that immunofluorescence performed with a Thr202/Tyr204-directed antibody provides a relative measure of pERK1/2 immunoreactivity but cannot resolve mono-phosphorylated from dual-phosphorylated ERK1/2 species or verify antibody specificity by molecular weight. Therefore, the CTCF values are reported as relative immunoreactivity rather than as absolute phosphoprotein quantification or a direct measure of ERK1/2 catalytic activity.

### 2.7. Statistical Analysis

GraphPad Prism (GraphPad Software, San Diego, CA, USA) was used for statistical analysis. For the MTT assay, the six technical replicate wells for each condition were averaged within each independent experiment, and the resulting per-experiment values were analysed using repeated-measures one-way ANOVA followed by Tukey’s multiple-comparisons test. Flow-cytometry population percentages were analysed using two-way ANOVA followed by Tukey’s multiple-comparisons test. For the TUNEL analysis, the per-experiment values were analysed using repeated-measures one-way ANOVA followed by Tukey’s multiple-comparisons test. For the immunofluorescence analyses, the per-experiment values were analysed using repeated-measures one-way ANOVA followed by Tukey’s multiple-comparisons test. Every treated group was compared with the untreated control as well as with the other treated groups, and the results of the relevant pairwise comparisons are reported in Section 3. All pairwise comparisons were two-sided, and adjusted *p*-values below 0.05 were considered statistically significant. Descriptive data are presented as mean ± standard deviation (SD). The statistical test applied to each assay is specified above and in the corresponding figure legend. Given the small number of independent biological replicates, all *p*-values should be regarded as exploratory, and no correction for multiple testing across assays was applied.

The replication structure was as follows. All experiments were repeated in three independent experiments (*n* = 3) performed on separate days using independently seeded cultures. For the MTT assay, each treatment condition included six technical replicate wells within each independent experiment. For flow cytometry, at least 1 × 10^4^ events were acquired for each analysed sample in each of the three independent experiments; acquired events were treated as observational measurements rather than as independent replicates. For the TUNEL analysis, five randomly selected microscopic fields were quantified within each independent experiment. For immunofluorescence analysis, at least 50 cells per group were analysed within each independent experiment. The acquired events, microscopic fields and quantified cells were considered technical or observational measurement units rather than independent biological replicates. Accordingly, the values obtained from these technical or observational units were first averaged within each independent experiment, and the resulting per-experiment means were used as the unit of statistical analysis, so that the independent experiment—rather than the individual well, event, microscopic field or cell—defined the *n* of every comparison reported here. Because this yields a small number of independent observations per group, the statistical findings should be interpreted as exploratory in vitro results, and the conclusions were limited to associations observed under the present experimental conditions.

## 3. Results

### 3.1. Effects of Fluoxetine on Metabolic Viability in Ishikawa and HDF Cells

**Figure 1:**
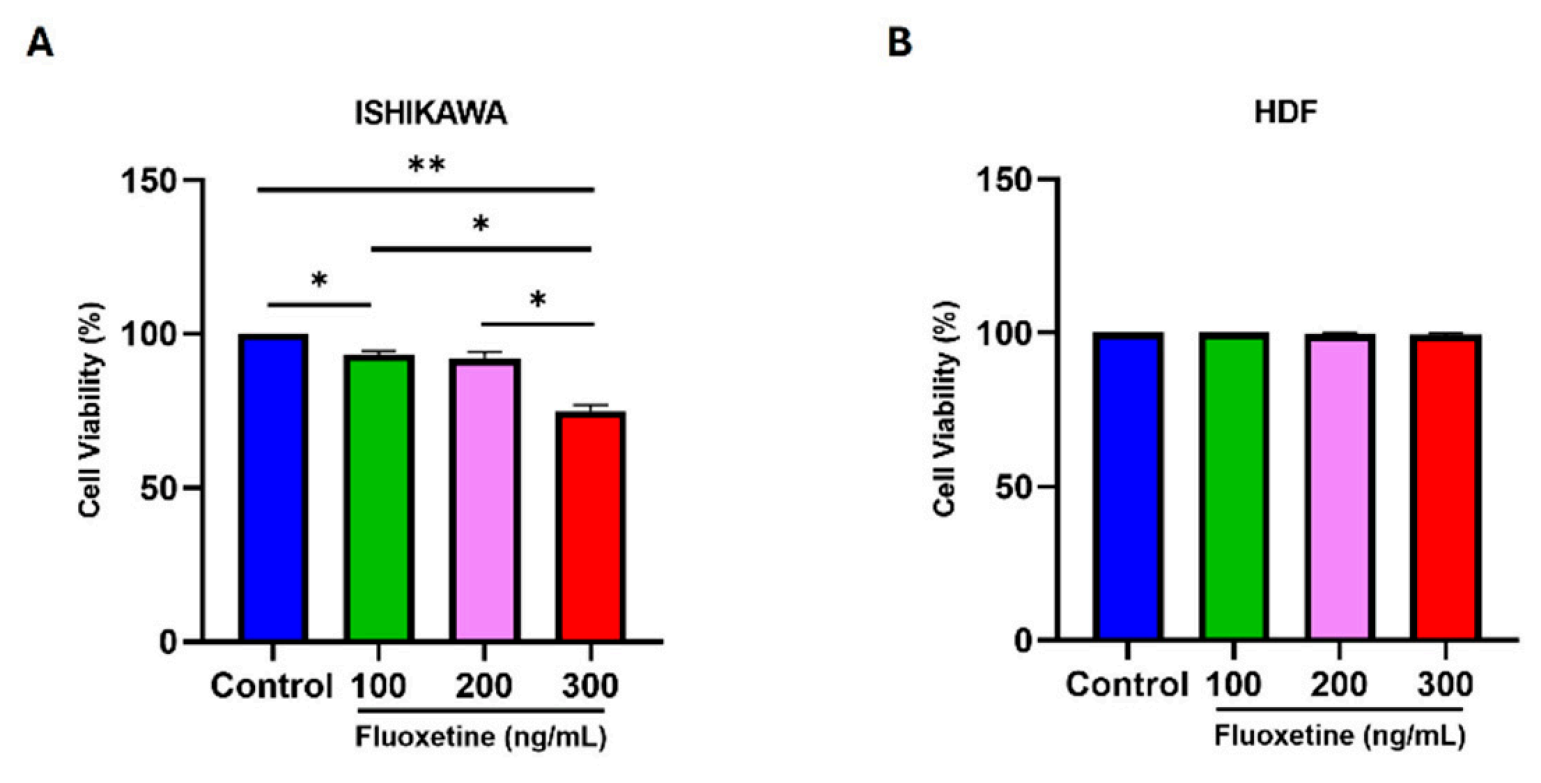
Effect of fluoxetine on metabolic viability in Ishikawa (**A**) and HDF (**B**) cells. Cells were treated with fluoxetine at 100, 200 and 300 ng/mL for 48 h. Viability is expressed as a percentage relative to untreated control cells. Data are presented as mean ± SD of three independent experiments (*n* = 3), each including six technical replicate wells per condition. Technical replicate values were averaged within each independent experiment before statistical analysis. Statistical comparisons were performed using repeated-measures one-way ANOVA followed by Tukey’s multiple-comparisons test. Only statistically significant pairwise comparisons are indicated on the graphs. * *p* < 0.05; ** *p* < 0.01. Exact adjusted *p*-values, including non-significant comparisons, are reported in Section 3.1.

In Ishikawa cells, fluoxetine reduced metabolic viability, with the most pronounced reduction observed at 300 ng/mL (Figure 1A). Viability was approximately 93.3%, 92.0% and 75.0% at 100, 200 and 300 ng/mL, respectively. Compared with untreated control cells, viability was significantly reduced at 100 ng/mL (adjusted *p* = 0.0246) and 300 ng/mL (adjusted *p* = 0.0054), whereas the reduction at 200 ng/mL did not reach statistical significance (adjusted *p* = 0.0500). No statistically significant difference was detected between the 100 and 200 ng/mL groups (adjusted *p* = 0.4061). Viability at 300 ng/mL was significantly lower than that at both 100 ng/mL (adjusted *p* = 0.0132) and 200 ng/mL (adjusted *p* = 0.0116). The 300 ng/mL concentration was estimated to correspond approximately to the IC_25_ level.

In HDF cells, metabolic viability remained preserved following fluoxetine exposure (Figure 1B). The values obtained for the untreated control and 100 ng/mL groups were identical, and no statistically significant differences were detected between the control and the 200 ng/mL (adjusted *p* = 0.7683) or 300 ng/mL (adjusted *p* = 0.4061) groups. No significant differences were identified among the fluoxetine-treated groups (all adjusted *p* > 0.05).

These findings indicate that fluoxetine exposure was associated with reduced metabolic viability in Ishikawa cells within the selected concentration range, whereas metabolic viability was largely preserved in HDF cells.

### 3.2. Annexin V–FITC/PI-Defined Cell Death Patterns and Microscopic Morphology

**Figure 2:**
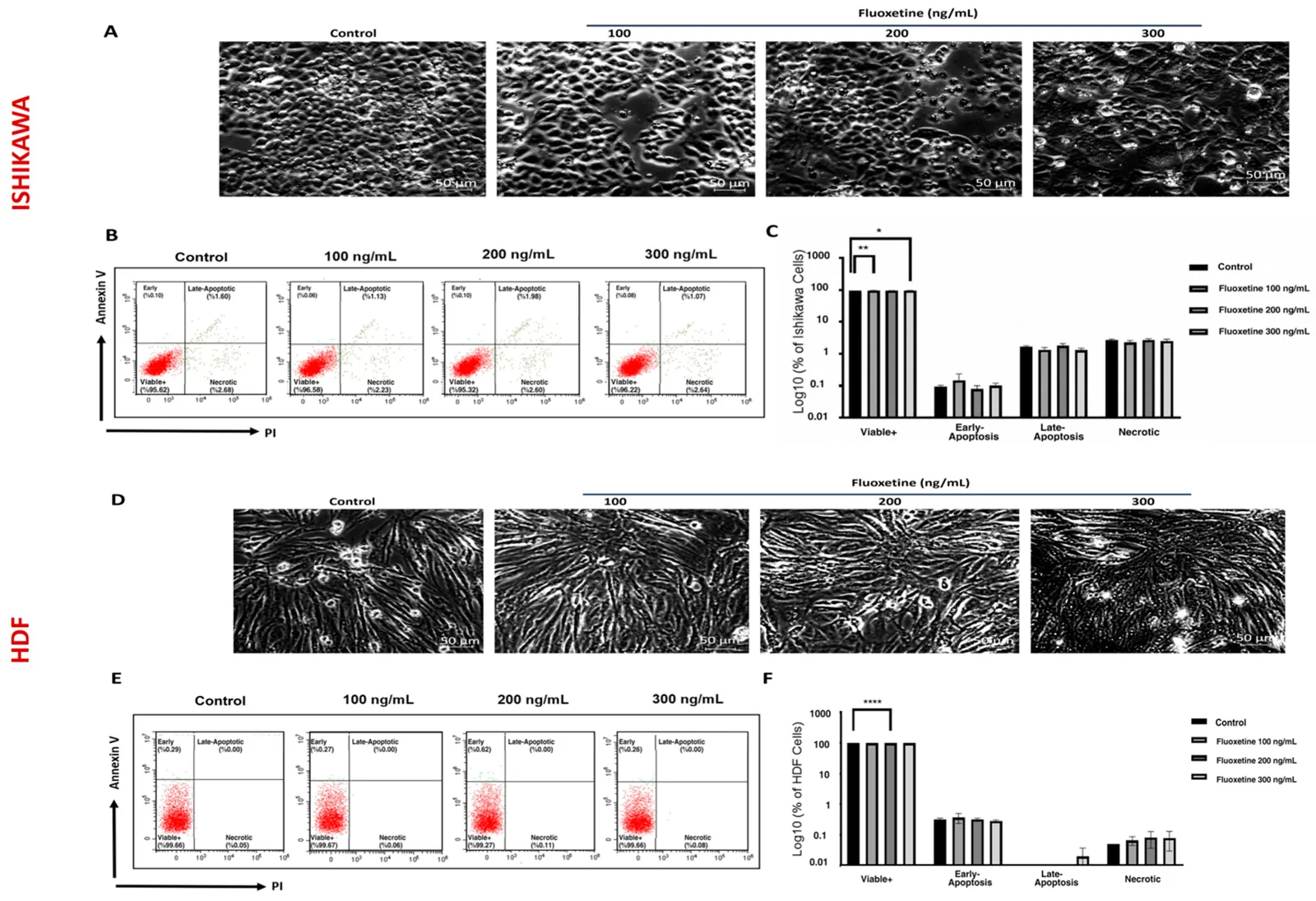
Fluoxetine-associated cell death-related patterns in Ishikawa and HDF cells assessed by annexin V–FITC/PI flow cytometry and morphology. (**A**–**C**) Ishikawa endometrial cancer cells; (**D**–**F**) human dermal fibroblasts (HDFs). (**A**,**D**) Representative phase-contrast images of cells in the control and fluoxetine-treated groups (100, 200 and 300 ng/mL) after 48 h; brightness and contrast were adjusted identically within each cell-line image set for display purposes to improve the visibility of the morphological changes. Scale bar: 50 µm. (**B**,**E**) Representative annexin V–FITC/PI dot plots, reproduced at an enlarged size so that the quadrant percentages are legible, showing quadrant-based classification of viable (annexin V^−^/PI^−^), early apoptotic (annexin V^+^/PI^−^), late apoptotic/secondary necrotic (annexin V^+^/PI^+^) and PI-positive/primary necrotic or membrane-compromised (annexin V^−^/PI^+^) cell populations. (**C**,**F**) Quantification of cell population distribution expressed as percentage of total cells and displayed on a log10 scale. Data are presented as mean ± SD from three independent experiments (*n* = 3). For each sample, ≥1 × 10^4^ events were acquired. Flow-cytometry population percentages were analysed using two-way ANOVA followed by Tukey’s multiple-comparisons test. Statistical significance is indicated as follows: * *p* < 0.05, ** *p* < 0.01, and **** *p* < 0.0001.

In Ishikawa cells, representative dot plots showed that the viable-cell fraction (annexin V^−^/PI^−^) remained high across all conditions, ranging from approximately 95.32% to 96.58% (Figure 2B). Early apoptotic cells (annexin V^+^/PI^−^) remained at very low levels (approximately 0.06–0.10%), whereas late apoptotic/secondary membrane-compromised cells (annexin V^+^/PI^+^) ranged from approximately 1.07% to 1.98%. The annexin V^−^/PI^+^ population ranged from approximately 2.23% to 2.68%. Quantitative analysis of three independent experiments showed small differences in the viable-cell fraction, with higher values at 100 ng/mL (*p* < 0.01) and 300 ng/mL (*p* < 0.05) compared with the untreated control (Figure 2C). No statistically significant treatment-related differences were indicated for the early apoptotic, late apoptotic or annexin V^−^/PI^+^ populations. At the single 48 h endpoint examined, the annexin V^−^/PI^+^ fraction was numerically higher than the early apoptotic annexin V^+^/PI^−^ fraction. Because earlier time points and apoptosis-specific markers were not assessed, this distribution does not establish the predominant mode or temporal sequence of cell death.

In HDF cells, representative dot plots showed that the viable-cell fraction (annexin V^−^/PI^−^) remained highly preserved across all conditions, ranging from approximately 99.27% to 99.67% (Figure 2E). Early apoptotic cells (annexin V^+^/PI^−^) were observed at low levels (approximately 0.26–0.62%), the annexin V^−^/PI^+^ population remained limited (approximately 0.05–0.11%), and late apoptotic/secondary membrane-compromised cells (annexin V^+^/PI^+^) were negligible. Quantitative analysis identified a statistically significant difference in the viable-cell fraction between the untreated control and 100 ng/mL groups (*p* < 0.0001; Figure 2F); however, the absolute numerical difference was minimal, and viability remained approximately 99% in all groups. No statistically significant treatment-related differences were indicated for the early apoptotic, late apoptotic or annexin V^−^/PI^+^ populations. Overall, the flow cytometry findings did not indicate marked cell death-related changes in HDF cells under the tested conditions.

In parallel with the flow cytometry findings, cellular morphological changes associated with fluoxetine treatment were evaluated microscopically. Untreated Ishikawa cells exhibited typical epithelial-like morphology, distinct cell–cell junctions, and high confluence. In groups treated with fluoxetine, a decrease in confluence, rounding and shrinking of cells, reduced surface attachment and increased cellular debris were observed (Figure 2A). In contrast, after fluoxetine application in HDF cells, the cells largely retained their typical spindle-shaped fibroblastic morphology, and cell adhesion and overall cellular integrity were not visibly impaired (Figure 2D). When these morphological observations were evaluated together with the cell population distributions obtained by flow cytometry, they indicated that treatment-related changes were more pronounced in Ishikawa cells.

### 3.3. Fluoxetine-Associated DNA Fragmentation

**Figure 3:**
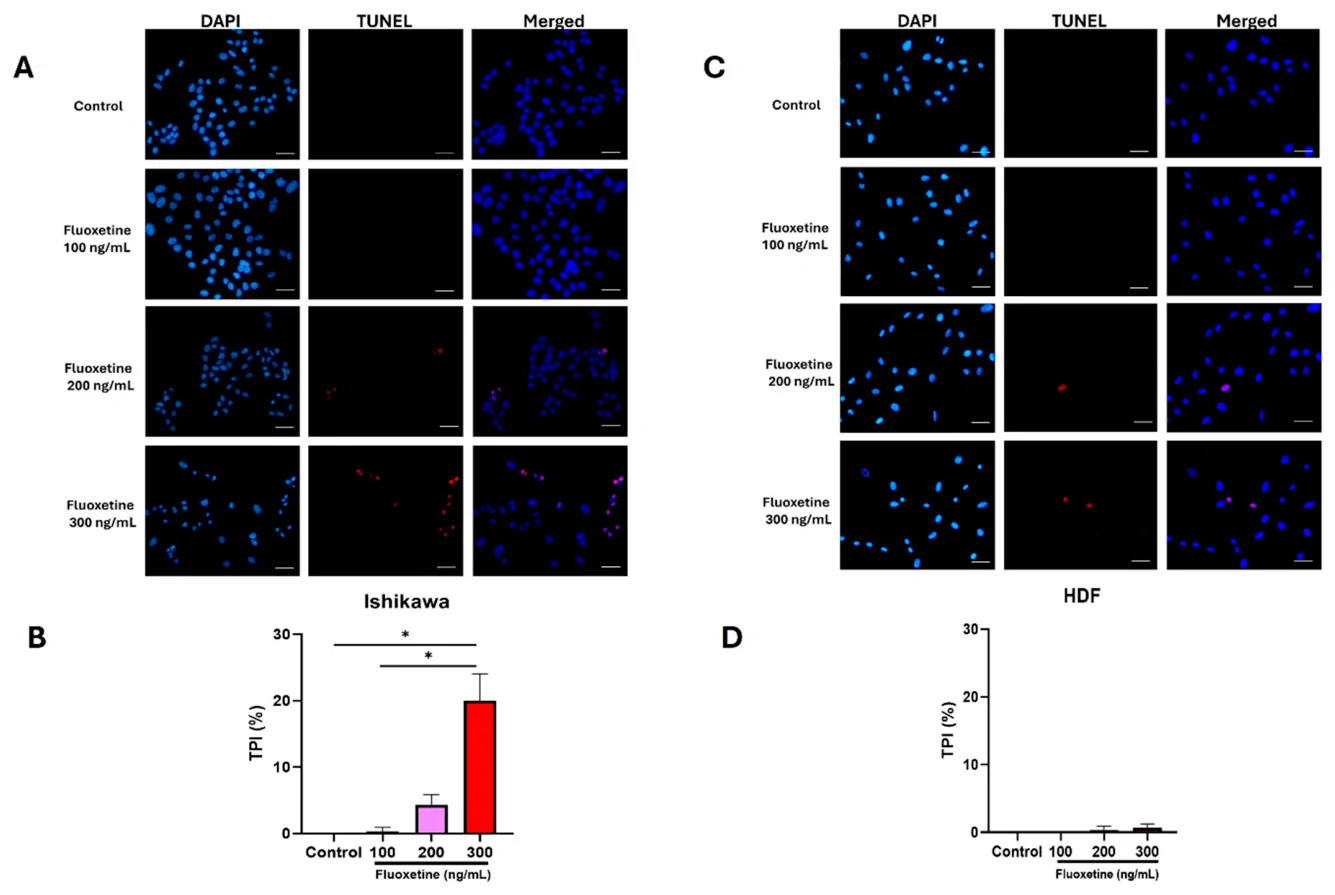
Fluoxetine-associated DNA fragmentation assessed by TUNEL. (**A**,**C**) Representative fluorescence images of untreated and fluoxetine-treated cells: nuclei are shown with DAPI (blue), and TUNEL positivity is shown in red; merged images are presented. Images were acquired using identical exposure settings within each cell-line image set, and the display brightness of the red channel was adjusted identically across the corresponding panels to improve visibility. (**B**,**D**) Quantitative analysis expressed as the TUNEL positivity index (TPI, %), defined as (number of TUNEL-positive nuclei/total number of DAPI-positive nuclei) × 100. TPI rather than an apoptotic index is reported because TUNEL positivity is not specific to apoptosis and may also label necrotic and late-apoptotic nuclei. Data are presented as mean ± SD from three independent experiments (*n* = 3). Five randomly selected microscopic fields were quantified within each independent experiment, and the resulting per-experiment values were used as the units of statistical analysis. Statistical comparisons were performed using repeated-measures one-way ANOVA followed by Tukey’s multiple-comparisons test. Only statistically significant pairwise comparisons are indicated on the graphs; exact adjusted *p*-values, including non-significant comparisons, are reported in Section 3.3. * *p* < 0.05. Scale bars = 100 µm.

Following fluoxetine administration to Ishikawa cells, TUNEL positivity, expressed as TPI (%), showed a concentration-related numerical increase, with the most pronounced elevation observed at 300 ng/mL (Figure 3A,B). Quantitative analysis showed that the TPI did not differ significantly from that of untreated control cells at 100 ng/mL (adjusted *p* = 0.7683) or 200 ng/mL (adjusted *p* = 0.0954). In contrast, the TPI was significantly higher at 300 ng/mL than in the untreated control (adjusted *p* = 0.0325) and the 100 ng/mL group (adjusted *p* = 0.0343). The difference between the 200 and 300 ng/mL groups did not reach statistical significance (adjusted *p* = 0.0833) (Figure 3B).

In HDF cells, TUNEL positivity remained very low under all experimental conditions (Figure 3C,D). The control and 100 ng/mL groups had identical per-experiment values, and no statistically significant differences were detected between the control and the 200 ng/mL (adjusted *p* = 0.7683) or 300 ng/mL (adjusted *p* = 0.4061) groups. No significant differences were identified among the fluoxetine-treated groups (all adjusted *p* > 0.05).

### 3.4. Fluoxetine-Associated Changes in Total ERK1/2 and pERK1/2 Immunoreactivity

**Figure 4:**
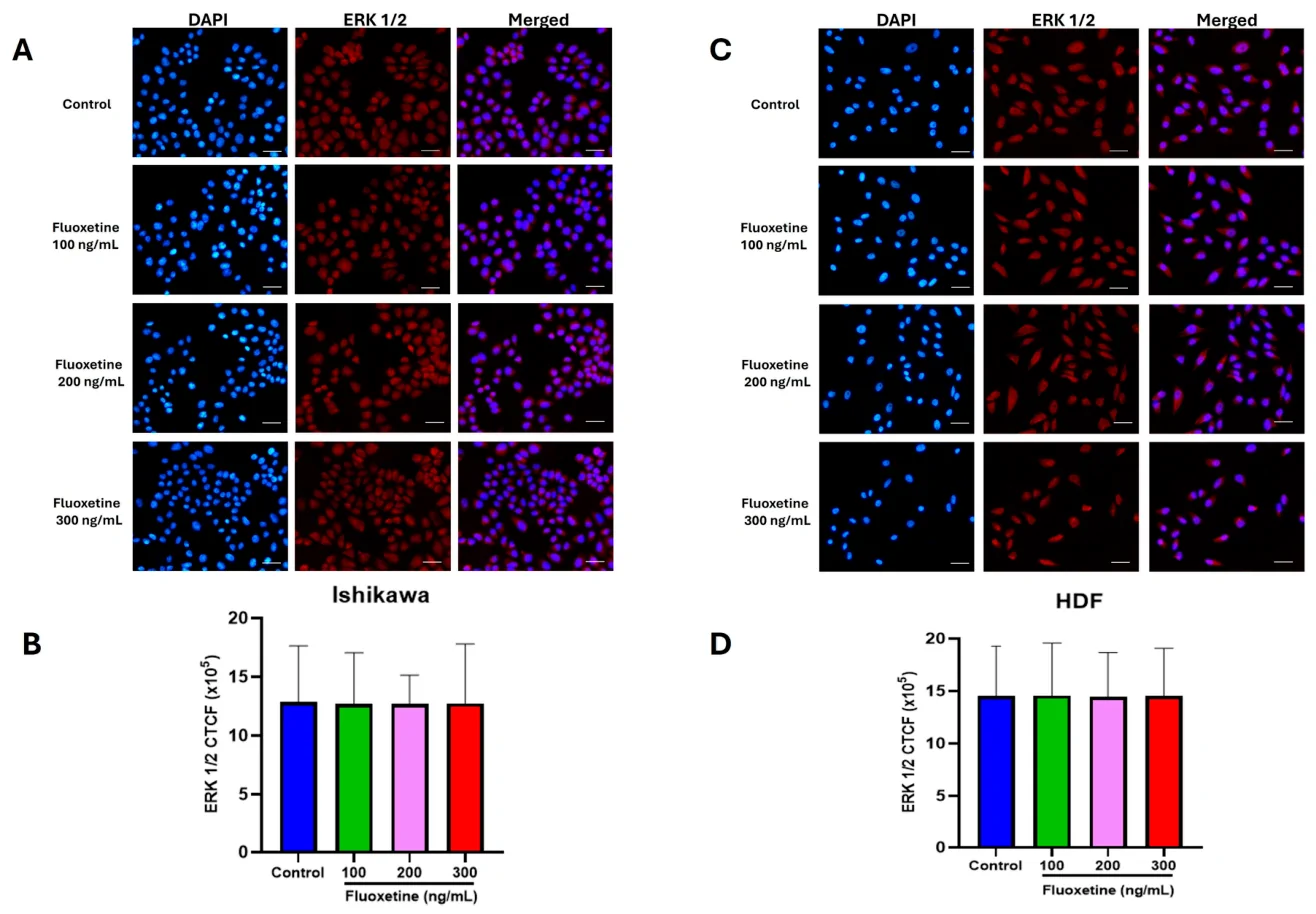
Total ERK1/2 immunofluorescence analysis in Ishikawa and HDF cells following fluoxetine treatment. (**A**,**C**) Representative fluorescence images of control and fluoxetine-treated groups: nuclei are shown with DAPI (blue), total ERK1/2 immunoreactivity is shown in red, and merged images are presented. (**B**,**D**) Quantitative analysis of total ERK1/2 immunoreactivity expressed as corrected total cell fluorescence (CTCF). Data are presented as mean ± SD from three independent experiments (*n* = 3). At least 50 cells per group were analysed within each independent experiment, and the resulting per-experiment means were used as the units of statistical analysis. Statistical comparisons were performed using repeated-measures one-way ANOVA followed by Tukey’s multiple-comparisons test. No statistically significant pairwise differences were detected. Scale bars = 100 µm.

Total ERK1/2 CTCF values remained comparable among the control and fluoxetine-treated groups in both Ishikawa and HDF cells. No statistically significant pairwise differences were detected in either Ishikawa cells (all Tukey-adjusted *p* ≥ 0.1408) or HDF cells (all Tukey-adjusted *p* ≥ 0.0914; Figure 4).

**Figure 5:**
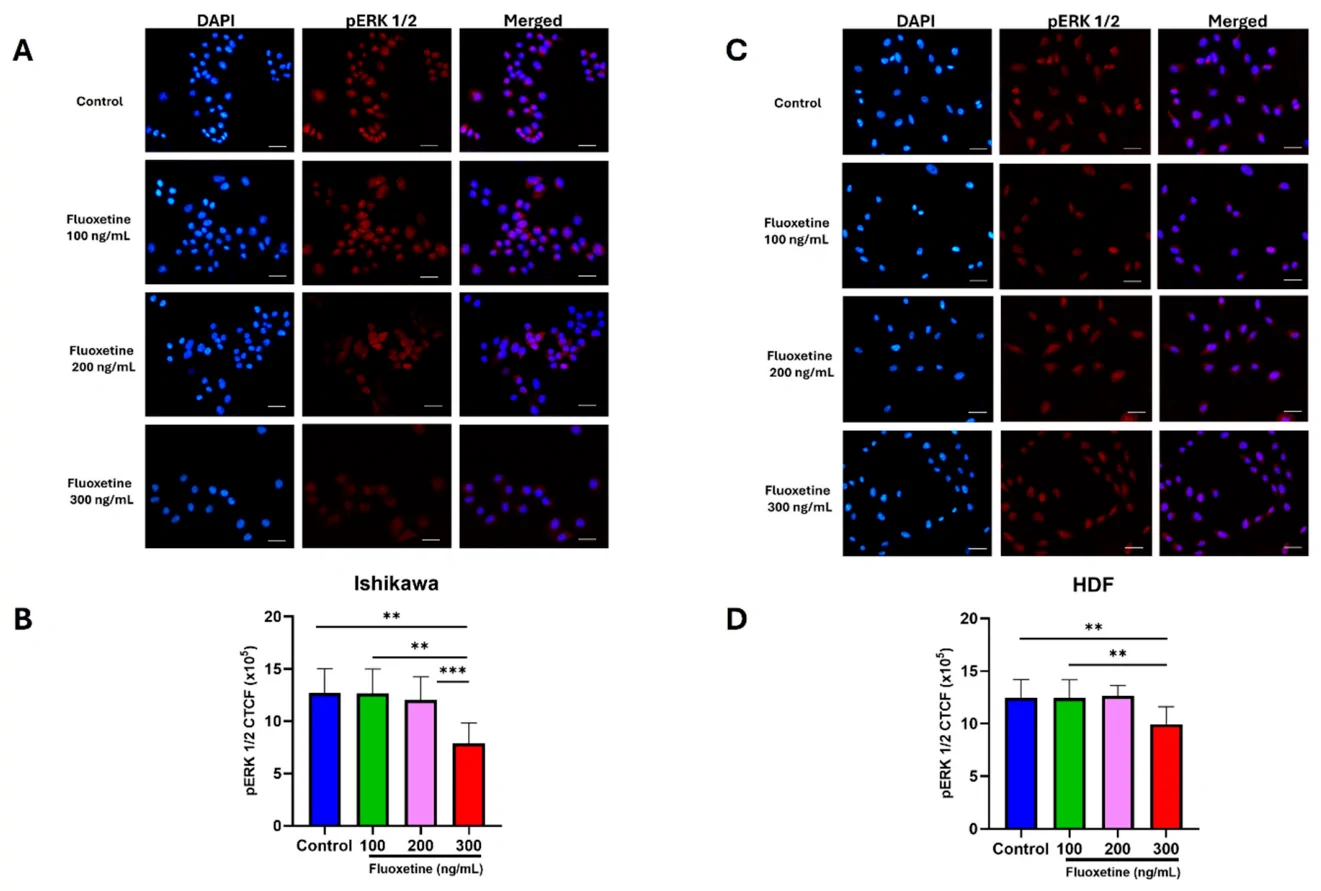
Immunofluorescence analysis of phosphorylated ERK1/2 (pERK1/2) in Ishikawa and HDF cells following fluoxetine treatment. (**A**,**C**) Representative fluorescence images of control and fluoxetine-treated groups: nuclei are shown with DAPI (blue), pERK1/2 (Thr202/Tyr204) immunoreactivity is shown in red, and merged images are presented. (**B**,**D**) Quantitative analysis of pERK1/2 immunoreactivity expressed as corrected total cell fluorescence (CTCF). At least 50 cells per group were analysed within each of three independent experiments, and the resulting per-experiment means were used as the units of statistical analysis. Data are presented as mean ± SD from three independent experiments (*n* = 3). Statistical comparisons were performed using repeated-measures one-way ANOVA followed by Tukey’s multiple-comparisons test. Only statistically significant pairwise comparisons are indicated on the graphs. ** *p* < 0.01 and *** *p* < 0.001. Scale bars = 100 µm.

In Ishikawa cells, pERK1/2 CTCF values remained comparable among the control, 100 ng/mL and 200 ng/mL groups (all Tukey-adjusted *p* > 0.05). At 300 ng/mL, pERK1/2 immunoreactivity was significantly lower than in the control (adjusted *p* = 0.0060), 100 ng/mL (adjusted *p* = 0.0061) and 200 ng/mL groups (adjusted *p* = 0.0010; Figure 5A,B). These findings indicate that exposure to 300 ng/mL fluoxetine was associated with reduced pERK1/2 immunoreactivity in Ishikawa cells, whereas total ERK1/2 immunoreactivity remained unchanged.

In HDF cells, pERK1/2 CTCF values did not differ significantly among the control, 100 ng/mL and 200 ng/mL groups (all Tukey-adjusted *p* > 0.05). At 300 ng/mL, pERK1/2 immunoreactivity was significantly lower than in the control (adjusted *p* = 0.0058) and 100 ng/mL groups (adjusted *p* = 0.0052), whereas the difference between the 200 and 300 ng/mL groups was not statistically significant (adjusted *p* = 0.1380; Figure 5C,D). Total ERK1/2 immunoreactivity remained unchanged across the HDF groups.

## 4. Discussion

The present study investigated the effects of fluoxetine on cellular viability, cell death-related phenotypes, DNA integrity and MAPK/ERK signalling in Ishikawa endometrial cancer cells using concentrations selected with reference to reported plasma exposure ranges, with human dermal fibroblasts (HDFs) as a non-malignant comparator. The principal findings were that fluoxetine exposure was associated with reduced metabolic viability, increased DNA fragmentation and decreased pERK1/2 immunoreactivity without a comparable alteration in total ERK1/2 immunoreactivity in Ishikawa cells, although the statistical patterns varied according to concentration and endpoint. Flow cytometry showed predominantly viable populations and low early apoptotic, late apoptotic and annexin V^−^/PI^+^ fractions in both cell types, without statistically significant treatment-related differences in the cell death-associated populations. Because flow cytometry was performed at a single 48 h endpoint, earlier transient changes in these populations cannot be excluded.

An important methodological aspect of this study is the dose selection strategy. Many in vitro studies investigating repurposed psychotropic drugs employ supraphysiological micromolar concentrations that primarily induce non-specific cytotoxicity and membrane rupture, thereby obscuring regulated signalling events [22]. In contrast, the two-stage dose optimisation applied here—beginning with a broad screening and subsequently narrowing the experimental window to ng/mL concentrations aligned with reported steady-state human plasma concentrations after chronic dosing—allowed the evaluation of biologically relevant stress responses rather than catastrophic cell death [23]. Under these conditions, Ishikawa cells exhibited a gradual, concentration-dependent decline in metabolic viability, while HDF cells largely preserved viability. Although MTT primarily reflects mitochondrial metabolic activity rather than direct cell number, the reduction in MTT signal together with elevated TUNEL staining indicates that fluoxetine exposure was associated with metabolic compromise and increased DNA fragmentation in Ishikawa cells at the tested plasma exposure-referenced concentrations [24,25]. Although the selected concentrations were chosen with reference to reported plasma exposure ranges, direct translational interpretation requires caution. Plasma concentrations do not necessarily reflect the biologically active drug exposure at the cellular level, because free drug availability, serum protein binding, intracellular accumulation and drug distribution may differ substantially between in vivo and in vitro conditions. In addition, fluoxetine is metabolised to norfluoxetine, an active metabolite that may contribute to biological effects in vivo but was not evaluated in the present experimental system. Therefore, the current concentration range should be interpreted as plasma exposure-referenced rather than as a direct surrogate for clinical tissue-level drug exposure.

Analysis of cell death-related populations by annexin V–FITC/PI staining showed that both Ishikawa and HDF cells remained predominantly viable after 48 h of fluoxetine exposure. No statistically significant treatment-related differences were detected in the early apoptotic (annexin V^+^/PI^−^), late apoptotic/secondary membrane-compromised (annexin V^+^/PI^+^) or annexin V^−^/PI^+^ populations. Although small statistically significant differences were identified in the viable-cell fraction at selected concentrations, the absolute numerical differences were limited and were not accompanied by corresponding increases in the cell death-associated populations. In the representative Ishikawa dot plots, the annexin V^−^/PI^+^ fraction was numerically higher than the early apoptotic fraction; however, this distribution does not establish either the predominant mode or the temporal sequence of cell death. The annexin V^−^/PI^+^ quadrant may also be influenced by cellular debris or by loss of surface phosphatidylserine epitopes during sample processing and therefore warrants cautious interpretation [26]. Moreover, because only a single 48 h endpoint was examined, an earlier transient annexin V^+^/PI^−^ population cannot be excluded. Clarification of the temporal sequence and molecular nature of the observed cellular responses will require time-course experiments incorporating earlier endpoints together with apoptosis-specific measurements, such as active (cleaved) caspase-3 staining or caspase-3/7 activity assays [27].

TUNEL analysis provided complementary evidence that fluoxetine exposure was associated with increased DNA strand breaks in Ishikawa cells, whereas HDF cells remained largely unaffected. Although TUNEL is widely used to detect DNA fragmentation, TUNEL positivity is not specific to apoptosis and may also occur during other forms of cell death [20]. In addition, the TUNEL assay has limited accuracy in differentiating necrotic cells from late-apoptotic cells [21]. Therefore, the increase in TUNEL positivity, together with reduced metabolic viability, indicates that fluoxetine exposure was associated with DNA fragmentation and metabolic compromise in Ishikawa cells. However, the absence of corresponding treatment-related changes in the early apoptotic, late apoptotic and annexin V^−^/PI^+^ populations means that the present data do not establish a specific mode of cell death. Although heterogeneous and overlapping regulated celldeath programmes have been described in cancer cell populations [25,28], the current findings are insufficient to determine whether any such programme was activated in this experimental model. Recent studies in gynaecological cancer models further emphasise that cell-death regulation and therapeutic vulnerability are highly pathway- and context-dependent. Apoptosis-related regulatory mechanisms have been investigated in epithelial ovarian cancer under hypoxic conditions, while targeted molecular and nanoparticle-based strategies, including Mcl1-directed genome editing in cervical cancer and TP53 restoration in ovarian cancer, have been explored as pathway-focused therapeutic approaches. These studies collectively support the need for cancer-type-specific and mechanism-oriented validation when interpreting drug-associated effects on cell fate and survival signalling [29,30,31].

An important observation of this study is the reduction in phosphorylated ERK1/2 immunoreactivity in Ishikawa cells exposed to 300 ng/mL fluoxetine, while total ERK1/2 immunoreactivity showed no comparable change. Because ERK1/2 phosphorylation is widely used as a canonical activation readout of the MAPK/ERK cascade, this pattern may reflect an alteration in ERK-related signalling status rather than generalised protein loss or staining artefact [11,32]. ERK signalling is a key node in endometrial cancer biology, integrating growth factor- and hormone-related inputs and stress-adaptation cues that collectively support proliferation and treatment resistance [33]. Consistent with this, ERK1/2 activation has been directly linked to pro-proliferative signalling in endometrial carcinoma models under cytokine stimulation [34]. In this context, the observed reduction in pERK1/2 immunoreactivity may be biologically relevant to stress-associated cellular responses; however, in the absence of pathway modulation experiments, it should not be interpreted as direct evidence that ERK suppression drives the observed reduction in metabolic viability or increase in DNA fragmentation [35]. A further methodological caveat must be emphasised. Immunofluorescence performed with a Thr202/Tyr204-directed antibody cannot distinguish mono-phosphorylated from dual-phosphorylated ERK1/2 species, which are known to coexist and to differ in catalytic competence [12], and it cannot formally exclude cross-reactivity of the total ERK1/2 antibody, since band identity and molecular weight cannot be verified without immunoblotting. The change reported here is therefore a change in pERK1/2 immunoreactivity, and it should not be equated with quantitative inhibition of ERK1/2 catalytic activity; Western blot confirmation of the pERK1/2-to-total-ERK1/2 ratio remains necessary before any firmer statement can be made.

The present data do not establish a causal relationship between reduced pERK1/2 immunoreactivity and the observed changes in metabolic viability or DNA fragmentation. Nevertheless, the parallel decrease in pERK1/2 signal, metabolic activity and DNA integrity suggests that fluoxetine exposure is associated with alterations in survival-related signalling in Ishikawa cells [36]. In hormonally responsive Ishikawa cells, which show oestrogen-driven proliferative responses and ERα-associated phenotypic features, changes in ERK1/2 activity may be biologically relevant in the context of cellular stress responses [37]. Given that aberrant MAPK/ERK-related signalling is implicated in endometrial cancer progression and resistance biology, the observed reduction in pERK1/2 immunoreactivity may indicate a shift in signalling status under fluoxetine exposure [33]. However, because ERK pathway inhibition, activation/rescue experiments, upstream MEK modulation, Western blotting and qPCR-based downstream target analyses were not performed, these findings should be interpreted as associative rather than as evidence of a confirmed causal mechanism.

Comparison with HDF cells provides additional insight into the biological context of these findings. Although decreased pERK1/2 immunoreactivity was also detected in HDF cells at the highest tested fluoxetine concentration, this change was not accompanied by a marked reduction in metabolic viability, increased DNA fragmentation or marked changes in the cell death-associated populations examined. This divergence is consistent with the concept that malignant cells often operate under chronic oncogenic and metabolic stress, becoming highly dependent on stress-adaptive and pro-survival signalling circuits [38]. In contrast, non-malignant cells can better buffer partial pathway perturbations and may be less reliant on any single survival pathway (the non-oncogene addiction framework) [39]. Given the signalling plasticity of dermal fibroblasts in maintaining tissue homeostasis [40], reduced pERK1/2 immunoreactivity may be tolerated without phenotypic collapse, even though ERK signalling broadly supports growth and survival programmes [36]. Thus, the differential outcomes observed here likely reflect context-dependent signalling vulnerability rather than true drug selectivity.

Taken together, these findings suggest that exposure to 300 ng/mL fluoxetine was associated with reduced pERK1/2 immunoreactivity, decreased metabolic activity and increased DNA fragmentation in Ishikawa endometrial cancer cells. However, annexin V/PI flow cytometry did not identify corresponding treatment-related increases in the early apoptotic, late apoptotic or annexin V^−^/PI^+^ populations at the single 48 h endpoint examined. Therefore, the combined findings demonstrate metabolic and DNA integrity-related changes but do not establish a specific mode or temporal sequence of cell death. This lack of concordance between the assays may reflect differences in the biological processes measured and the timing of their detection, reinforcing the need for time-course and apoptosis-specific analyses [26,41].

Several limitations of this study should be acknowledged, and they define the boundaries within which the present findings can be interpreted. First, and most importantly, ERK1/2 and pERK1/2 levels were assessed by immunofluorescence only and were not independently validated by Western blotting or other quantitative protein-based methods. Immunofluorescence with a phospho-specific antibody reports relative immunoreactivity, cannot resolve mono- from dual-phosphorylated ERK1/2 species, and cannot verify antibody specificity by molecular weight; the ERK1/2-related findings are therefore reported as associative changes in immunoreactivity rather than as demonstrated pathway inhibition. Second, qPCR analyses of ERK pathway-related downstream transcriptional targets (for example DUSP6, EGR1 and FOS) and of genes involved in fluoxetine and norfluoxetine disposition were not performed, so the transcriptional consequences of the observed signalling change remain unknown. Third, causal involvement of ERK signalling in the observed phenotypes was not directly tested using pharmacological or genetic modulation, such as MEK/ERK inhibition with U0126 or PD98059, ERK activation/rescue experiments, or upstream MEK modulation; consequently, no causal claim is made, and the parallel changes reported here are interpreted explicitly as associations. Fourth, only a single exposure duration of 48 h was studied. Because annexin V/PI profiles are strongly time-dependent, the low early apoptotic fraction observed may reflect the timing of the measurement rather than a true absence of apoptosis; time-course experiments combined with cleaved caspase-3, cleaved PARP, caspase activity assays, LDH release, mitochondrial membrane potential analysis and necroptosis-related markers are required to define the mode of cell death more precisely. Fifth, the number of independent experiments was limited. Although the values obtained from technical and observational units were averaged within each independent experiment so that the independent experiment served as the unit of analysis, the number of independent observations was small, and the cell-to-cell variability of the immunofluorescence measurements was high. The statistical findings should therefore be regarded as exploratory rather than as confirmatory evidence, and the study was not powered to exclude modest effects at the tested concentrations. Sixth, the findings are restricted to a single endometrial cancer cell line and to one non-malignant cell type of non-endometrial origin. Confirmation in additional malignant models, such as HEC-1A and RL95-2 cells, and in non-malignant endometrial epithelial or stromal cells is required before any statement about cancer-specific selectivity or tissue relevance can be made; the HDF comparison presented here indicates only that the concentrations used are tolerated by a representative non-malignant cell type. Finally, the chemical stability of fluoxetine and its residual concentration in culture medium over the 48 h exposure period were not verified analytically; therefore, the administered concentrations should be interpreted as nominal initial concentrations. The active metabolite norfluoxetine was also not evaluated. Taken together, these limitations mean that the present work should be regarded as an exploratory, hypothesis-generating study; the use of plasma exposure-referenced concentrations and the integrated phenotypic and signalling readouts provide a defined starting point for the confirmatory experiments outlined above.

## 5. Conclusions

The present findings suggest that exposure to 300 ng/mL fluoxetine was associated with reduced metabolic viability, increased DNA fragmentation and decreased pERK1/2 immunoreactivity in Ishikawa endometrial cancer cells under the in vitro conditions examined. Annexin V/PI flow cytometry did not identify corresponding treatment-related increases in the early apoptotic, late apoptotic or annexin V^−^/PI^+^ populations at the single 48 h endpoint, and the present data therefore do not establish a specific mode or temporal sequence of cell death. The comparatively limited phenotypic changes observed in HDF cells should be interpreted as the response of a general non-malignant comparator rather than as direct evidence of endometrial cancer-specific selectivity. Because the ERK1/2-related observations were based on immunofluorescence alone, no pathwaymodulation experiments were performed, and only one malignant cell line and one exposure duration were examined, these findings do not establish therapeutic efficacy or a causal role for ERK1/2 signalling. The results should be regarded as exploratory and hypothesis-generating. Confirmation using Western blotting and qPCR, MEK/ERK inhibition and rescue experiments, time-course analyses incorporating apoptosis-specific markers, and additional malignant and non-malignant endometrial cell models is required before translational interpretation can be considered.

## Data Availability

The data presented in this study are available on request from the corresponding author. No new genetic or sequencing data were generated or deposited in a public database as part of the present study. The cell stocks used originated from the commercial collections specified in Section 2.1 (ECACC 99040201, RRID:CVCL_2529; ATCC PCS-201-012).

## Abbreviations

AKT, protein kinase B; BSA, bovine serum albumin; CTCF, corrected total cell fluorescence; DAPI, 4′,6-diamidino-2-phenylindole; DMEM, Dulbecco’s Modified Eagle Medium; DMSO, dimethyl sulfoxide; DNA, deoxyribonucleic acid; dUTP, deoxyuridine triphosphate; ECACC, European Collection of Authenticated Cell Cultures; EDTA, ethylenediaminetetraacetic acid; ERK1/2, extracellular signal-regulated kinase 1/2; FBS, foetal bovine serum; FITC, fluorescein isothiocyanate; HDF, human dermal fibroblast; IC_25_, 25% inhibitory concentration; IC_50_, half-maximal inhibitory concentration; IgG, immunoglobulin G; LDH, lactate dehydrogenase; MAPK, mitogen-activated protein kinase; MEK, mitogen-activated protein kinase kinase; MTT, 3-(4,5-dimethylthiazol-2-yl)-2,5-diphenyltetrazolium bromide; NF-κB, nuclear factor kappa B; PARP, poly(ADP-ribose) polymerase; PBS, phosphate-buffered saline; pERK1/2, phosphorylated extracellular signal-regulated kinase 1/2; PI, propidium iodide; PI3K, phosphoinositide 3-kinase; qPCR, quantitative polymerase chain reaction; RPMI-1640, Roswell Park Memorial Institute 1640 medium; SD, standard deviation; SSRI, selective serotonin reuptake inhibitor; STAT3, signal transducer and activator of transcription 3; TdT, terminal deoxynucleotidyl transferase; TEY, threonine–glutamate–tyrosine motif; TPI, TUNEL positivity index; TUNEL, terminal deoxynucleotidyl transferase dUTP nick-end labelling.
